# Association of altered plasma long-chain fatty acids with migraine-related disability: a clinical cross-sectional study

**DOI:** 10.3389/fnut.2026.1893087

**Published:** 2026-07-15

**Authors:** Fang Liu, Xiran Yu, Lu Ning, Na Li, Zhangqin Yang, Jinyi Tian, Hamza Khan, Ge Tan

**Affiliations:** 1 Department of Neurology, The First Affiliated Hospital of Chongqing Medical University, Chongqing, China; 2 Department of Neurology, Qianjiang Central Hospital of Chongqing, Chongqing, China; 3 Migraine Diagnosis and Treatment Center, The First Affiliated Hospital of Chongqing Medical University, Chongqing, China

**Keywords:** arachidonic acid, disability burden, fatty acid metabolism, migraine, palmitic acid, stearic acid

## Abstract

**Objective:**

The relationship between migraine and lipid metabolism, as well as its underlying mechanisms, remains poorly defined. In lipid metabolism, long-chain fatty acids (LCFAs) play a role in regulating cellular energy homeostasis and inflammation. Emerging evidence suggests a link between LCFAs and migraine pathophysiology. We investigated the association between altered plasma LCFAs and migraine-related disability.

**Methods:**

In this cross-sectional study, plasma samples and clinical data were collected from 118 individuals with migraine, comprising 79 with episodic migraine (EM) and 39 with chronic migraine (CM), and 118 matched healthy controls. Concentrations of 13 plasma LCFAs were quantified using targeted gas chromatography–mass spectrometry. Associations with migraine phenotype and clinical burden were assessed using regression and correlation analyses.

**Results:**

Plasma levels of palmitic acid (C16:0), stearic acid (C18:0), and arachidonic acid (C20:4 n-6) were significantly higher in migraine patients than in controls, whereas docosahexaenoic acid (C22:6 n-3) levels were significantly lower. Multivariate logistic regression identified C16:0, C18:0, and C20:4 n-6 as independent correlates of migraine (AUC = 0.912). Notably, higher levels of C16:0 and C20:4 n-6 were independently associated with with CM relative to EM (AUC = 0.762), and positively correlated with monthly headache days, acute medication use, and disability scores.

**Conclusion:**

Our study showed that increased concentrations of plasma LCFAs, specifically C16:0, C18:0, and C20:4 n-6, were independently associated with migraine. C16:0 and C20:4 n-6 were associated with the burden of migraine-related disability. These findings suggest a potential role for the regulation of lipid metabolism in the management and prevention of migraine.

## 1 Introduction

Migraine is a common neurological disorder characterized by recurrent episodes of unilateral throbbing headache (1). According to the 2021 Global Burden of Neurological Disorders Study, approximately 1.16 billion individuals (14.0% of the global population) are affected by migraine, making it the leading cause of disability among people aged 20–59 years (2). Current migraine diagnosis primarily relies on structured clinician-patient interviews based on established diagnostic criteria (1, 3); however, significant heterogeneity persists in clinical manifestations, treatment responses, and comorbid symptoms (4), underscoring the urgent need to identify effective diagnostic biomarkers to improve early diagnosis and therapeutic strategies (5).

Migraine is considered a disorder of energy metabolism, and lipid metabolism, as a critical component of energy homeostasis, may contribute to its pathogenesis (6). Fatty acids, as core components of lipid metabolism, play essential roles in maintaining normal physiological function (7, 8). Fatty acid metabolic dysregulation is implicated in various central nervous system disorders, including alzheimer’s disease, parkinson’s disease, and multiple sclerosis (9, 10). In migraine, abnormalities in fatty acid and lipid metabolism have been observed in the plasma and cerebrospinal fluid (11). In 2019, a Dutch study analyzed plasma metabolite levels from 2,800 migraine patients using ^1^H-NMR spectroscopy and found altered high-density lipoprotein (HDL) metabolism and lower omega-3 levels in male patients (12). In 2021, an extensive genome-wide association study (GWAS) on the genetic overlap between blood metabolites and migraine analyzed 972 blood metabolites and identified 44 metabolites that were significantly genetically associated with migraine, of which 36 were lipids. Notably, palmitic acid and phosphatidylinositol showed higher genetic overlap with migraine, whereas lower levels of docosahexaenoic acid (DHA) appeared to be genetically protective (13). Furthermore, multiple randomized controlled trials have demonstrated that supplementation with eicosapentaenoic acid (EPA) and DHA significantly reduces the number of monthly headache days in migraine patients (14–16). Long-chain fatty acids (LCFAs), defined as fatty acids with a carbon chain length ≥ 14, are involved in physiological processes such as energy metabolism, structural maintenance, and signaling regulation in various neurological disorders (17–20). These findings suggest that an imbalance in fatty acid metabolism may contribute to the pathogenesis of migraine, and supplementation with specific fatty acid ratios could benefit migraine treatment. However, variations in inclusion/exclusion criteria and analytical methods across studies have limited the clinical applicability of fatty acids as reliable biomarkers or predictors for early migraine diagnosis.

To address this gap, we quantified plasma levels of LCFAs in healthy controls (HC) and migraine patients using targeted gas chromatography–mass spectrometry (GC–MS) and evaluated their associations with clinical features and disease burden.

## 2 Methods

### 2.1 Participants

**Figure 1:**
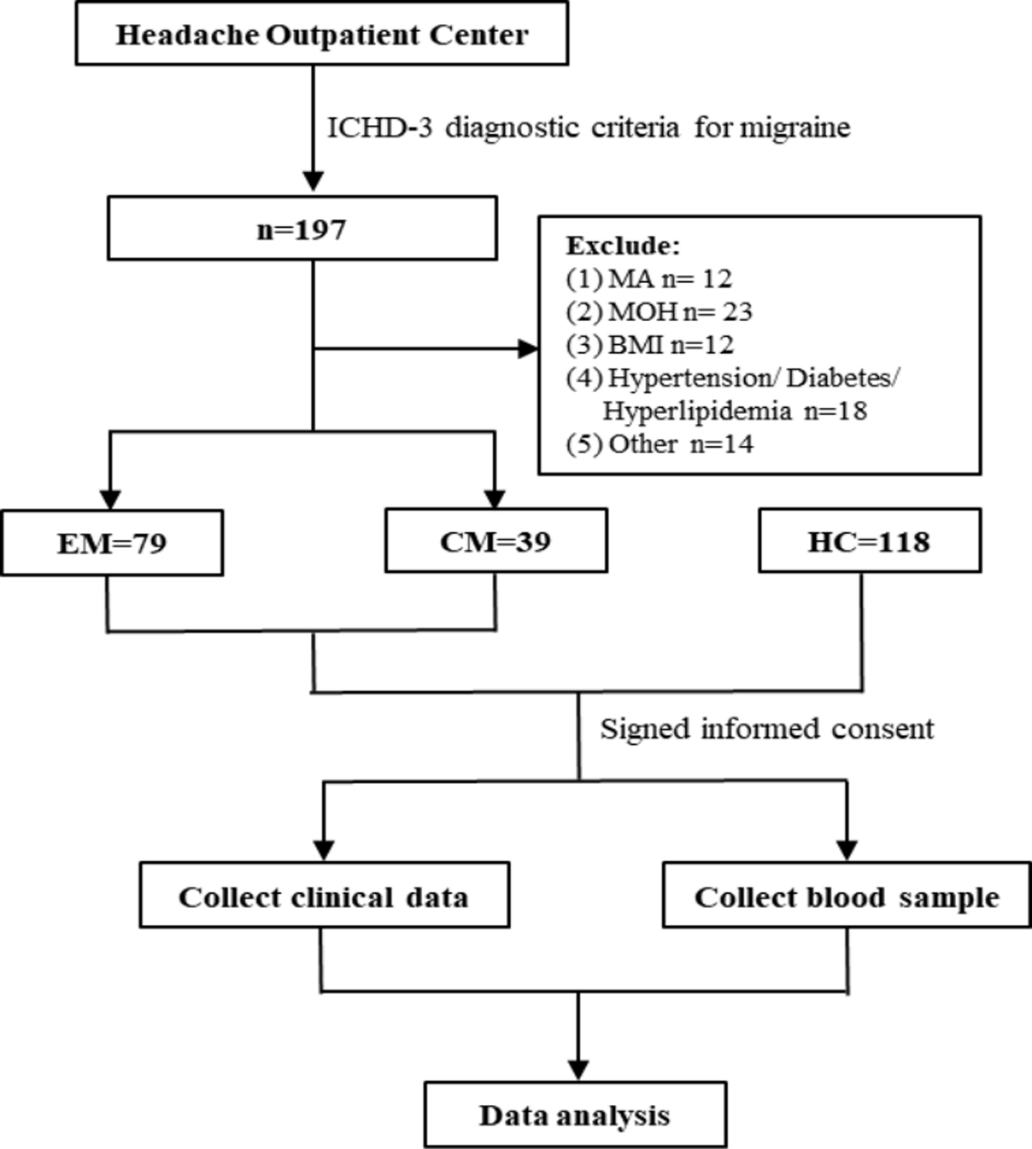
Research process flowchart.

The study recruited 118 migraine patients from the headache clinic of the First Affiliated Hospital of Chongqing Medical University between January 2024 and June 2024, along with 118 matched health controls (HC). Inclusion criteria for migraine patients were as follows: (1) age 18–60 years; (2) diagnosis of episodic migraine (EM) without aura or chronic migraine (CM) according to ICHD-3 criteria (1); (3) no regular orally administered prophylactic medication within the past month; and (4) signed informed consent. In this study, regular prophylactic medication was defined as either daily administration of conventional preventive agents or every-other-day administration of novel small-molecule calcitonin gene-related peptide (CGRP) receptor antagonists (21). Exclusion criteria included: (1) other primary or secondary headaches per ICHD-3 criteria, including but not limited to migraine with aura and medication-overuse headache; (2) primary hypertension, type 2 diabetes, hyperlipidemia, atherosclerotic cardiovascular/cerebrovascular diseases; (3) body mass index (BMI) < 18.5 kg/m^2^ or > 28.0 kg/m^2^ (22); (4) Mental disorders diagnosed by DSM-V; and (5) cognitive impairment (Mini-Mental State Examination score ≤27). The study protocol was approved by the Ethics Committee of The First Affiliated Hospital of Chongqing Medical University and registered with the Chinese Clinical Trial Registry (ChiCTR2500098619). A brief flowchart of patient recruitment is shown in Figure 1.

### 2.2 Clinical characteristics

We collected clinical data on gender, age, and -BMI- from both migraine patients and healthy controls. Additionally, migraine-specific clinical features, such as duration of migraine, monthly headache days (MHD), numerical rating scale (NRS) score, and monthly acute medication days (AMD), were recorded. Disability burden in migraine patients was assessed using Migraine Disability Assessment (MIDAS) and Headache Impact Test (HIT-6), while anxiety and depression were evaluated via the Hospital Anxiety and Depression Scale (HADS). After data collection, all participants underwent standardized neurological examinations.

### 2.3 Measurement of plasma fatty acid concentrations

#### 2.3.1 Sample collection

During the headache interictal phase and morning fasting period, venous blood samples were collected from the median cubital vein using 5 mL EDTA-anticoagulant tubes. Within 30 min of collection, the plasma was separated by centrifugation at 3000 × g for 15 min at 4 °C and then stored at −80 °C in an ultra-low temperature freezer.

#### 2.3.2 Sample pretreatment

Sample preparation and derivatization were performed according to the methyl chloroformate (MCF) method described by Smart et al. (23). First, 150 μL of plasma was added to a microcentrifuge tube. Subsequently, 50 μL of 4 mol/L NaOH, 4 μL of internal standard (Stearic acid-d35), and 200 μL of methanol were added sequentially. The mixture was vortexed thoroughly and centrifuged at 12,000 rpm for 15 min at 4 °C. The supernatant was carefully transferred to a glass tube. Then, 34 μL of pyridine and 24 μL of a methanol-chloroform mixture were added, mixed and vortexed for 30 s. The addition of 24 μL of methanol-chloroform mixture was repeat. Next, 400 μL of saturated NaHCO₃ solution was added, vortexed for 10 s, and centrifuged at 2,000 rpm for 10 min at 4 °C. Finally, the lower chloroform layer was transfered to a microcentrifuge tube, dehydrated with sodium sulfate powder, and 100 μL of the liquid was collected into an autosampler vial. The laboratory technicians were blinded to the patient’s group allocation.

#### 2.3.3 Targeted GC–MS analysis

Fatty acids were analyzed using an Agilent Intuvo 9,000 gas chromatograph coupled with an MSD5977B mass spectrometer in electron ionization mode at 70 eV. A BD-1701 capillary column was employed. Samples were injected in pulsed splitless mode with an inlet temperature of 290 °C and a helium flow rate of 1 mL/min. The oven temperature program was initially held at 45 °C for 2 min, ramped at 9 °C/min to 180 °C (held for 5 min), then at 40 °C/min to 220 °C (held for 5 min), followed by 40 °C/min to 240 °C (held for 11.5 min), and finally 80 °C/min to 280 °C. Temperatures for the guard chip, auxiliary heating zone, quadrupole, and ion source were set to 280 °C, 250 °C, 230 °C, and 150 °C, respectively. Mass spectra were acquired in the 30–550 m/z range at a scan rate of 1.563 u/s with a solvent delay of 5.5 min.

### 2.4 Data analysis

Fatty acid chromatographic peak heights were extracted using Agilent ChemStation software (v2.6) and corrected using internal standards. Absolute concentrations were calculated based on calibration curves established from fatty acid standards. Statistical analyses and visualizations were performed using SPSS 26.0 and GraphPad Prism 9.0. Data normality was assessed via the Shapiro–Wilk test, with results expressed as mean ± standard deviation or median (interquartile range, IQR). Two-group comparisons used independent samples t-tests or Mann–Whitney U tests. Categorical variables were analyzed using Chi-square tests. Logistic regression models were used to assess the diagnostic performance of fatty acids for migraine, and the receiver operating characteristic (ROC) curve were generated. Relationships between fatty acid levels and clinical features were evaluated via Spearman’s correlation, multiple linear regression, and partial correlation analyses. All tests were two-tailed, with a significance threshold of *p* < 0.05.

## 3 Results

### 3.1 Demographic and clinical characteristics

A total of 197 patients were initially recruited according to the inclusion criteria. After applying the exclusion criteria, 79 patients were removed for the following reasons: migraine with aura (*n* = 12), medication-overuse headache (*n* = 23), BMI < 18.5 or > 28.0 kg/m^2^ (*n* = 12), comorbidities including hypertension, diabetes mellitus, or hyperlipidemia (*n* = 18), and concurrent diseases such as hepatitis, tuberculosis, intracranial space-occupying lesions, chronic pain disorders, or other infectious conditions (*n* = 14). Consequently, 118 patients (79 episodic and 39 chronic migraine) were included in the final analysis (Figure 1), along with 118 age-matched healthy controls. The mean age was 37.19 ± 9.19 years for the migraine group and 37.71 ± 8.47 years for the HC. No statistically significant differences were observed in age (*p* = 0.648), BMI (*p* = 0.975), or gender distribution (*p* = 0.364) between the migraine and HC groups (Table 1). In comparisons between migraine subgroups, patients with CM had significantly higher monthly headache days (20.00 ± 15.00 vs. 5.00 ± 4.00, *p* < 0.001) and MIDAS scores (63.00 ± 45.00 vs. 11.00 ± 10.00, p < 0.001) compared with the EM group. The CM group also reported more AMD (9.00 ± 2.00 vs. 6.00 ± 5.00, *p* < 0.001) and higher usage of acute non-specific analgesics (7.33 ± 1.80 vs. 5.00 ± 3.00, *p* < 0.001). Other clinical features showed no significant differences between the migraine subgroups (*p* > 0.05) (Table 2).

**Table 1:** Demographic characteristics of healthy controls and migraine patients.

Characteristics	HC (*n* = 118)	Migraine (*n* = 118)	EM^bd^ (*n* = 79)	CM^cd^ (*n* = 39)	*p-*value^a^
Age (years)	37.71 ± 8.47	37.19 ± 9.19	37.01 ± 9.40	37.54 ± 8.88	0.648
BMI (kg/m^2^)	22.72 ± 2.13	22.71 ± 2.21	22.58 ± 2.19	22.96 ± 2.25	0.975
Female, *n* (%)	96 (81.36%)	100 (84.75%)	68 (86.07%)	32 (82.05%)	0.488

^a^Indicates Migraine group vs. HC group; ^b^indicates EM group vs. HC group; c indicates CM group vs. HC group; ^d^indicates EM group vs. CM group. *p* > 0.05, the difference is not statistically significant. For age (years), the *p*-values for groups ^b, c^, and ^d^ are 0.588, 0.913, and 0.772; for BMI (kg/m^2^), the corresponding *p*-values are 0.668, 0.538, and 0.381. For female, the p-values for groups ^b, c^, and ^d^ are 0.385, 0.923, and 0.567, respectively.

**Table 2:** Clinical characteristics of migraine patients.

Clinical characteristics	EM (*n* = 79)	CM (*n* = 39)	*p*-value
Duration of migraine (years)	7.00 ± 15.00	10.00 ± 15.00	0.112
Age at onset (years)	27.43 ± 8.06	26.77 ± 8.87	0.686
Monthly headache days (MHD)	5.00 ± 4.00	20.00 ± 15.00	<0.001
Nausea or/and vomiting	49(62.03%)	23(58.97%)	0.749
Photophobia and phonophobia	37(46.84%)	20(51.28%)	0.649
NRS score			
Headache of peak intensity	7.00 ± 3.00	8.00 ± 2.00	0.416
Headache of non-peak intensity	6.00 ± 3.00	6.33 ± 2.01	0.199
HIT-6	61.19 ± 6.00	61.41 ± 6.18	0.564
MIDAS	11.00 ± 10.00	63.00 ± 45.00	<0.001
HADS	8.03 ± 3.64	8.52 ± 2.60	0.382
HADS-A	4.00 ± 2.00	3.95 ± 2.03	0.890
HADS-D	4.00 ± 4.00	4.59 ± 1.62	0.199
Monthly acute medication intake days (AMD)	6.00 ± 5.00	9.00 ± 2.00	<0.001
Days of use acute-non specific analgesic	5.00 ± 3.00	7.33 ± 1.80	<0.001
Days of use acute-specific analgesic	2.00 ± 3.00	2.00 ± 3.00	0.514
Family history, *n* (%)	35 (44.30%)	19 (48.72%)	0.651

HIT-6, Headache Impact Test; MIDAS, Migraine Disability Assessment; NRS score, Numerical Rating Scale score; HADS, Hospital Anxiety and Depression Scale; AMD, Monthly cute medication intake days. *p* < 0.05 indicates statistical significance.

### 3.2 Comparison of fatty acid concentrations across groups

Significant differences were observed between the migraine and HC groups for C16:0 (palmitic acid, p < 0.001), C18:0 (stearic acid, p < 0.001), C20:4 n-6 (arachidonic acid, p < 0.001), and C22:6 n-3 (DHA, *p* = 0.007). Subgroup analyses revealed significant differences in these fatty acids when comparing each of the EM and CM groups with the HC group. Furthermore, the EM group showed significantly lower levels of C16:0 and C20:4 n-6 compared with the CM group (*p* < 0.05) (Table 3).

**Table 3:** Differences in fatty acid concentrations between healthy controls and migraine patients.

Fatty acid (μg/mL)	HC (*n* = 118)	Migraine (*n* = 118)	EM (*n* = 79)	CM (*n* = 39)	*p*-value^a^
C14:0	5.58 ± 1.66	5.98 ± 1.55	5.95 ± 1.49	6.03 ± 1.68	0.057
C15:0	4.11 ± 1.31	4.23 ± 1.54	4.22 ± 1.53	4.25 ± 1.57	0.521
C16:0	49.97 ± 10.58	73.83 ± 16.48	69.43 ± 13.51^bd^	82.75 ± 18.43^cd^	<0.001
C17:0	4.71 ± 1.42	4.73 ± 1.53	4.73 ± 1.65	4.73 ± 1.27	0.909
C18:0	14.38 ± 6.06	22.25 ± 6.98	21.53 ± 6.68^b^	23.73 ± 7.40^c^	<0.001
C18:1 n-9	11.57 ± 2.59	11.00 ± 3.39	11.10 ± 3.15	10.81 ± 3.87	0.150
C18:2 n-6	4.60 ± 0.93	4.56 ± 0.85	4.55 ± 0.78	4.58 ± 0.99	0.748
C18:3 n-3	3.86 ± 0.93	3.83 ± 0.99	3.85 ± 0.98	3.80 ± 1.02	0.823
C20:1 n-9	4.13 ± 1.31	4.05 ± 1.68	3.94 ± 1.68	4.27 ± 1.22	0.573
C20:3 n-6	4.13 ± 1.31	4.01 ± 1.40	3.95 ± 1.43	4.15 ± 1.33	0.506
C20:4 n-6	2.75 ± 1.06	3.66 ± 1.45	3.32 ± 1.40^bd^	4.34 ± 1.31^cd^	<0.001
C20:5 n-3	4.45 ± 1.26	4.26 ± 1.36	4.28 ± 1.32	4.22 ± 1.45	0.261
C22:6 n-3	4.03 ± 1.11	3.63 ± 1.14	3.68 ± 1.09^b^	3.53 ± 1.24^c^	0.007

^a^indicates Migraine group vs. HC group; ^b^indicates EM group vs. HC group; ^c^indicates CM group vs. HC group; ^d^indicates EM group vs. CM group. p < 0.05 shows statistical significance, and *p* < 0.05 for ^a–d^.

### 3.3 The ROC analysis of fatty acids for migraine diagnosis

**Figure 2:**
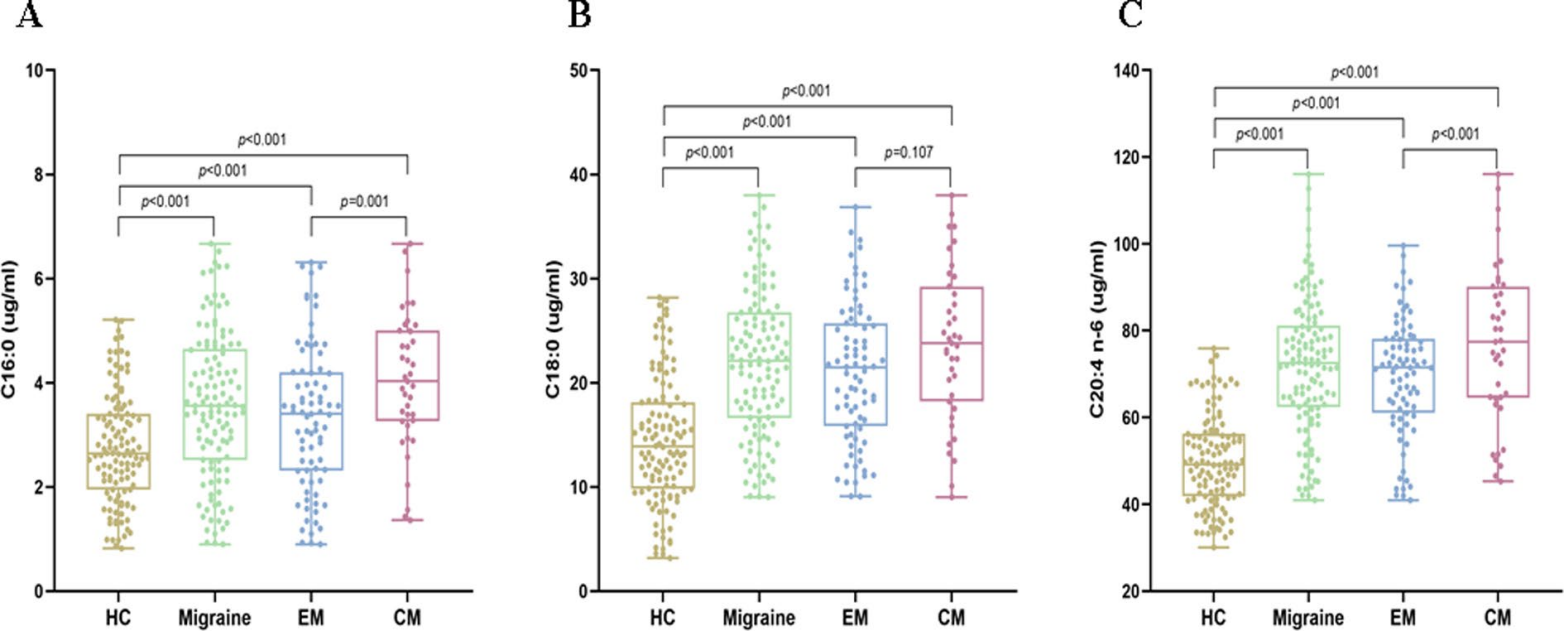
Inter-group distribution comparison of C16:0 **(A)**, C18:0 **(B)**, and C20:4 n-6 **(C)** in migraine subtypes and controls. *p* < 0.05 indicates statistical significance.

**Figure 3:**
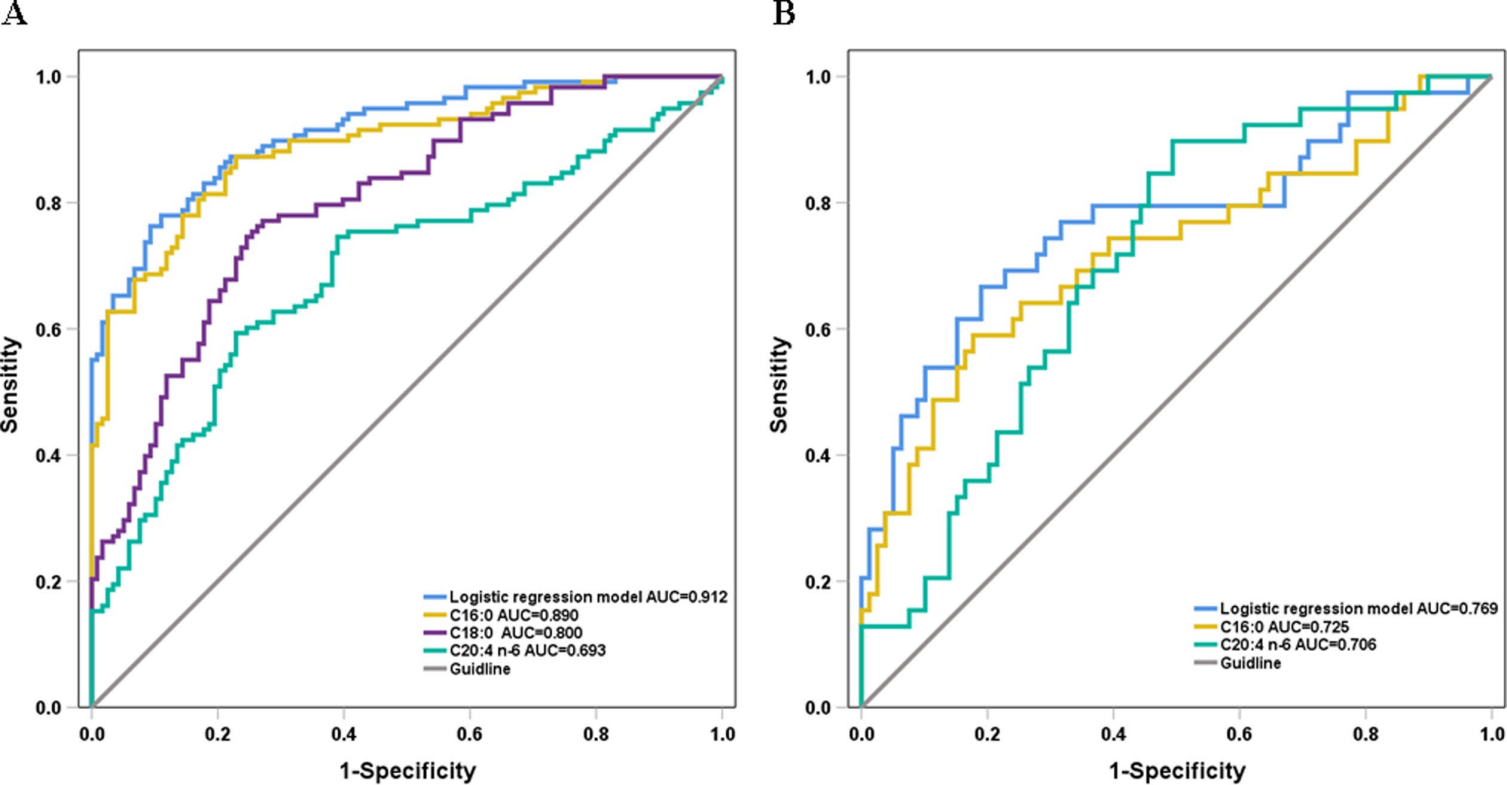
The ROC curve of plasma fatty acids for migraine classification. **(A)** AUC distinguishing migraine patients from healthy controls. **(B)** AUC distinguishing chronic migraineurs from episodic migraineurs.

After adjusting for covariates (age, sex and BMI), binary logistic regression identified elevated plasma levels of C16:0 (OR = 1.128, 95% CI 1.088–1.169, *p* < 0.001), C18:0 (OR = 1.095, 95%CI: 1.027–1.169, *p* = 0.006), and C20:4 n-6 (OR = 1.706, 95% CI: 1.237–2.353, *p* = 0.001) as independent factors for migraine (Table 4). The distributions of these three fatty acids in the HC group, the migraine group, and the subgroups are shown in Figure 2. The ROC curve constructed from the regression model for identifying migraine yielded an area under the curve (AUC) of 0.912 (95%CI, 0.877–0.947), indicating excellent discriminative ability. Individually, C16:0, C18:0, and C20:4 n-6 yielded AUCs of 0.890 (95% CI, 0.849–0.931), 0.800 (95% CI, 0.745–0.855), and 0.693 (95% CI, 0.625–0.761), respectively (Figure 3A; Supplementary Table S1).

**Table 4:** The binary logistic regression model of healthy controls and migraine patients.

Variables	*β*	SE	Wald value	*p-*value	OR	95%CI
Age	−0.016	0.023	0.462	0.496	0.984	0.941–1.030
BMI	0.039	0.094	0.172	0.678	1.040	0.864–1.251
Female	0.171	0.523	0.107	0.743	1.187	0.426–3.311
C16:0	0.120	0.018	43.578	<0.001	1.128	1.088–1.169
C18:0	0.091	0.033	7.618	0.006	1.095	1.027–1.169
C20:4 n-6	0.534	0.164	10.625	0.001	1.706	1.237–2.353

*p* < 0.05 indicates statistical significance.

Further analysis to differentiate EM from CM indicated that higher plasma levels of C16:0 (OR = 1.051, 95% CI: 1.020–1.084, *p* = 0.001) and C20:4 n-6 (OR = 1.582, 95% CI: 1.132–2.210, *p* = 0.007) were independently associated with CM relative to EM (Table 5). The AUC of this model was 0.769 (95% CI, 0.670–0.867) (Figure 3B; Supplementary Table S2).

**Table 5:** The binary logistic regression model of episodic migraineurs and chronic migraineurs.

Variables	*β*	SE	Wald value	*p*-value	OR	95%CI
Age	0.016	0.024	0.441	0.507	1.016	0.970–1.064
BMI	0.031	0.103	0.089	0.765	1.031	0.843–1.261
Female	−0.439	0.608	0.521	0.470	1.551	0.471–5.101
C16:0	0.500	0.016	10.355	0.001	1.051	1.020–1.084
C20:4 n-6	0.458	0.171	7.217	0.007	1.582	1.132–2.210

### 3.4 Relationship between fatty acids and migraine clinical characteristics

**Figure 4:**
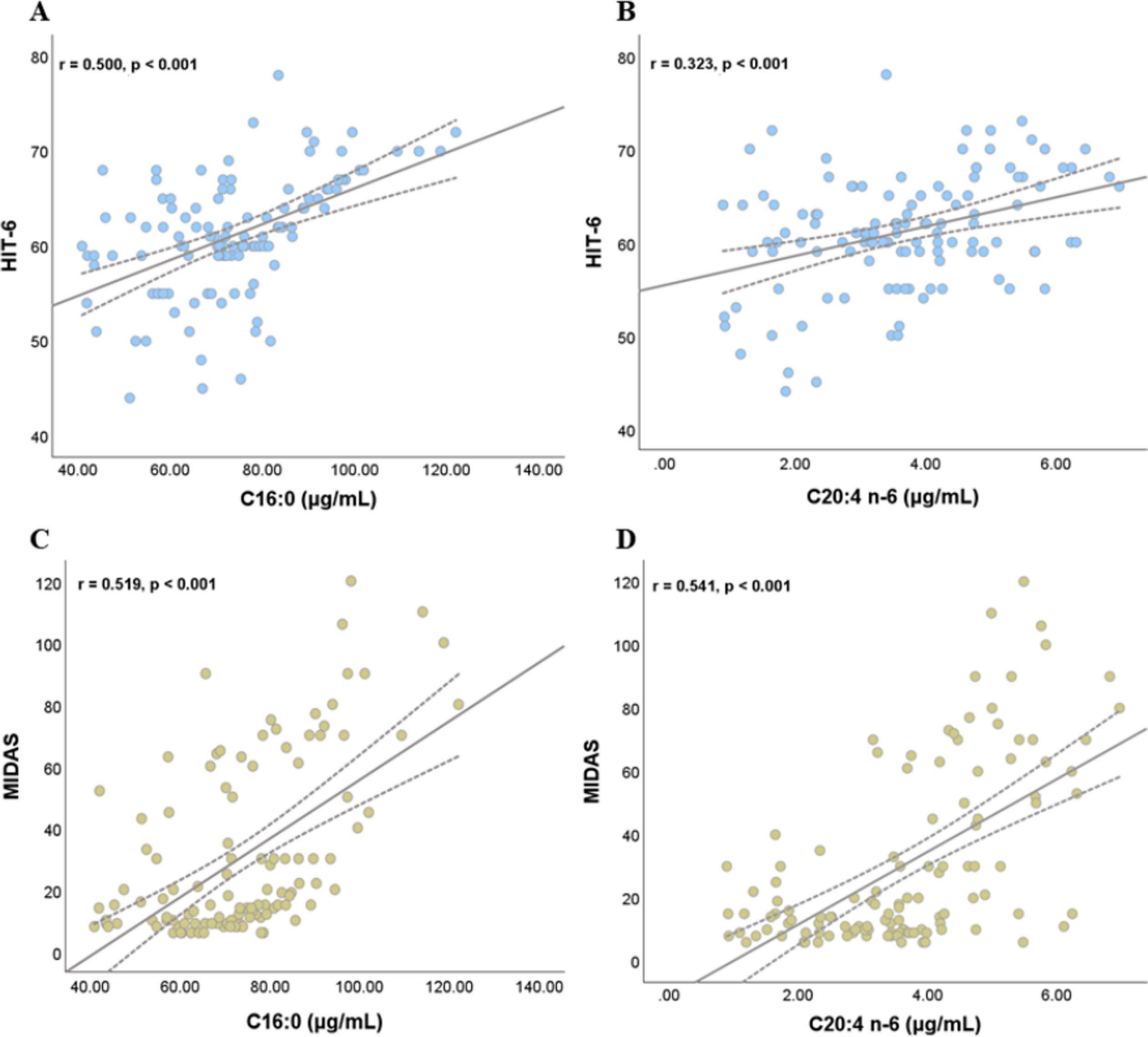
Spearman correlation between plasma fatty acids and HIT-6 or MIDAS scores. r represents the correlation coefficient, *p* < 0.05 denotes statistical significance.

Spearman correlation analysis showed that C16:0 (*r* = 0.500, *p* < 0.001) and C20:4 n-6 (*r* = 0.323, p < 0.001) were positively correlated with HIT-6. Similarly, MIDAS scores were positively correlated with C16:0 (*r* = 0.519, p < 0.001) and C20:4 n-6 (*r* = 0.541, p < 0.001) (Figure 4). After adjusting for age, sex, and BMI, multiple linear regression analysis consistently confirmed these associations (Supplementary Table S3). In addition, partial correlation analysis revealed that C16:0 was positively correlated with MHD (*p* = 0.002), while C20:4 n-6 was positively correlated with MHD (p < 0.001) and AMD (*p* = 0.005). Other clinical characteristics of migraine showed no significant correlation with either C16:0 and C20:4 n-6 (*p* > 0.05) (Table 6).

**Table 6:** Partial correlation analysis of fatty acids with clinical characteristics of migraine.

Clinical Characteristics	C16:0		C20:4 n-6	
	*r*	*p-*value	*r*	*p-*value
Duration of migraine	0.049	0.606	−0.032	0.731
Age at onset	−0.050	0.598	0.033	0.729
MHD	0.292	0.002	0.339	<0.001
Nausea or/and vomit	0.092	0.327	0.087	0.354
Photophobia and phonophobia	0.113	0.229	0.038	0.685
NRS score (peak intensity)	0.159	0.09	0.128	0.174
HADS	−0.076	0.419	0.142	0.129
AMD	0.059	0.528	0.263	0.005
Family history	0.000	0.999	−0.003	0.977

*r*, correlation coefficient; *p* < 0.05 indicates statistical significance.

## 4 Discussion

Migraine is a complex paroxysmal disorder involving disruptions in energy metabolism. Fatty acids, as key substrates and regulators of energy homeostasis, influence physiological balance through absorption, biosynthesis, and catabolism (24). This study examined the relationships between 13 plasma LCFAs and migraine-related clinical features. Migraine patients exhibited significantly altered plasma LCFA concentrations compared with HC. Considering the influence of age, gender, and BMI on both migraine pathogenesis (2) and fatty acid metabolism (25), logistic regression analyses identified plasma C16:0, C18:0, and C20:4 n-6 as significant independent predictors for migraine after adjusting for these covariates, with high diagnostic efficacy (AUC = 0.912).

Our results demonstrated that saturated fatty acids (SFAs), particularly C16:0 and C18:0, were elevated in the CM group. Chronic exposure to high concentrations of SFAs has been widely recognized as a risk factor for neurological and cardiovascular diseases, including Alzheimer’s disease, stroke, and cardiovascular diseases (26–29). C16:0, the most abundant SFA in humans (30), is synthesized from carbohydrate and amino acid metabolism or obtained from the diet (31, 32). C18:0 is primarily synthesized from the elongation of C16:0 in the endoplasmic reticulum or obtained directly from diet (33). The balance between C16:0 and C18:0 biosynthesis and degradation is crucial for maintaining lipid homeostasis. In humans, C16:0 and C18:0 exhibit a certain degree of tissue specificity. C16:0 is present at higher levels in subcutaneous adipose tissue but accumulates to a lesser extent in visceral fat, C18:0 shows the opposite pattern, with greater accumulation in visceral fat and cell membrane structures. C16:0 serves as a precursor for carbon chain elongation catalyzed by the elongation of very long chain fatty fcids enzyme family to produce longer-chain saturated fatty acids, including C18:0. Furthermore, it can be converted by enzymes such as stearoyl-CoA desaturase 1 (SCD1) into monounsaturated fatty acids. Both types of fatty acids can be desaturated by SCD1, albeit at markedly different rates: approximately 9.2% of C18:0 is converted to oleic acid (C18:1) via SCD1, whereas the conversion rate for C16:0 is only 3.9%. This difference underlies their distinct metabolic fates. Moreover, compared with C16:0, C18:0 is incorporated into plasma triglycerides and cholesteryl esters at a 30–40% lower rate, but into phosphatidylcholine at an approximately 40% higher rate (32, 33). This observation may also explain why a diet high in C18:0 leads to a more modest postprandial elevation of blood lipids (triglycerides), and some studies have even suggested that a diet high in C18:0 diet can lower low-density lipoprotein (LDL) while raising HDL in plasma, thereby exerting an anti-atherosclerotic effect (34, 35). Furthermore, in contrast to the lipotoxic effects of C16:0, in the nervous system, C18:0 protects cortical neurons from oxidative damage by modulating glutamate uptake and promoting the production of antioxidant enzymes under physiological conditions (36, 37). In contrast to the above findings, a diet high in C16:0 and C18:0 in a rat model of osteoarthritis increased bone pain sensitivity (38) and induced mechanical allodynia in wild-type rats (39). Together with our findings, these data suggest that the potential role of SFAs (especially C16:0 and C18:0) in migraine pathogenesis requires further investigation.

Our study also found significantly elevated plasma levels of C20:4 n-6 in patients with migraine. C20:4 n-6, also known as arachidonic acid (AA), is an n-6 essential fatty acid that serves as the direct precursor for bioactive eicosanoids (e.g., prostaglandins, leukotrienes, and thromboxanes) synthesized via cyclooxygenase, lipoxygenase, and cytochrome P450 enzymatic pathways (40). These eicosanoids of AA are generally pro-inflammatory and play a critical role in modulating the inflammatory balance (41). As early as 1968, Carlson et al. first discovered that intravenous prostaglandin E1 infusion induces migraine-like headaches in healthy subjects (42). Since then, many preclinical and clinical studies have shown that prostaglandins are involved in the pathogenesis of migraine (43–45). In 2022, Loonen et al. (46) found that cortical spreading depolarization in mice elevates circulating AA and anti-inflammatory lipids such as DHA, EPA, and DPA. Contrary to Loonen et al., the present study found significantly lower plasma DHA levels in migraine patients than in HCs, partially consistent with the GWAS by Tanha et al. (13). This discrepancy is understandable. As key membrane phospholipid components, AA and DHA are functionally synergistic yet antagonistic and metabolically competitive, regulating downstream mediator production via shared enzyme systems and maintaining a dynamic balance (47). In a migraine mouse model, elevated AA was accompanied by increased DHA and other anti-inflammatory lipid precursors, possibly reflecting a compensatory protective response. These findings suggest that exogenous DHA supplementation may reduce AA and its pro-inflammatory mediators during headache attacks, thereby preserving omega-3/omega-6 balance and alleviating migraine burden (15, 48). Future research could focus on the enzymes and proteins responsible for omega-3/omega-6 framework synthesis and degradation to identify novel therapeutic targets.

CM is highly disabling and impacts quality of life (49). Approximately 4.7% of EM patients transition to CM every year (50). Identifying risk factors for this transformation is therefore critical. In our study, plasma levels of C16:0 and C20:4 n-6 were significantly higher in CM than EM patients and served as independent predictors of chronicity. Moreover, both fatty acids were positively associated with MIDAS and HIT-6 scores, the two most widely used instruments to quantify migraine-related disability (51). These results suggest that higher levels of LCFAs may contribute not only to disease onset but also to disease burden and progression in migraine patients. The recent OVERCOME study highlighted MIDAS as a marker of migraine progression (50), emphasizing the importance of MIDAS in reflecting the disability burden of migraine. These findings further suggest that dysregulated fatty acid metabolism may serve as a potential correlate and predictive factor for migraine and its exacerbation.

The relationship between peripheral and central fatty acid concentrations remains an open question. Some studies have reported that the concentrations of plasma fatty acids are proportional to that of brain tissue (26, 52). In 2013, a study of healthy adults revealed significant positive correlations between total blood omega-3 levels and cerebrospinal fluid omega-3 subcomponents (DPA and DHA), with similar associations observed for PUFAs and AA (53). Interestingly, Naudí et al. (54) examined the concentrations of LCFAs in 12 brain regions of 28 healthy adults and found that the distribution of fatty acids was significantly region-specific, with a significant negative correlation of monounsaturated fatty acids and a significant positive correlation of PUFAs along the caudal-rostral axis. The brain efficiently synthesizes SFAs, but it has limited capacity for endogenous PUFA production and thus relies on peripheral circulation for precursors (55). Recent studies have confirmed that exogenous dietary SFAs can be transported to the brain via specialized pathways (56), and fatty acid transport proteins at the blood–brain barrier mediate dynamic exchanges between the peripheral and central nervous systems (57, 58). Together, these findings suggest that the levels of peripheral fatty acids in migraine patients may partially reflect cerebral fatty acid dynamics.

Our study also has several limitations. First, the cross-sectional design precludes causal inference. Longitudinal studies are needed to validate these associations. Second, dietary intake data were not collected; given that diet affects circulating fatty acid levels, future studies should incorporate detailed nutritional assessments. Third, we did not account for the potential effects of preventive medications, which vary in mechanism and may influence lipid metabolism differently. Furthermore, although we included patients who had not taken prophylactic agents regularly in the preceding month, the long-term medication history of these patients—particularly in those with chronic migraine—may still have influenced LCFA levels despite a 1-month washout period. Given that different prophylactic agents may exert distinct effects on lipid metabolism, further investigation into their relationship with LCFA is warranted. Lastly, during clinical sample collection, we did not explicitly define or restrict the specific time interval since the patient’s most recent headache episode, which may have introduced some degree of variability. These limitations will be addressed and, as far as possible, avoided in future studies.

## 5 Conclusion

In conclusion, this study identified elevated plasma levels of C16:0, C18:0, and C20:4 n-6 as potential independent associated factors for migraine. Among them, C16:0 and C20:4 n-6 were also associated with the burden of migraine-related disability. These findings suggest that dysregulated fatty acid metabolism may contribute to migraine pathophysiology and progression, warranting further mechanistic investigation.

## Data Availability

The original contributions presented in the study are included in the article/Supplementary material, further inquiries can be directed to the corresponding author.
