# Supplementary material for: Association of altered plasma long-chain fatty acids with migraine-related disability: a clinical cross-sectional study
Source: Front Nutr. 2026 Jul 15;13:1893087. doi: 10.3389/fnut.2026.1893087 (PMC13416853; doi:10.3389/fnut.2026.1893087)
Supplement: Supplementary file 1 [file Table_1.DOCX]

**Supplementary Materials**

**Table S1**

The ROC curve analysis of fatty acids for healthy controls and migraine patients.

| **ROC curve analysis** | **AUC** | **95% CI** |
| --- | --- | --- |
| Logistic regression model | 0.912 | 0.877-0.947 |
| C16:0 | 0.890 | 0.849-0.931 |
| C18:0 | 0.800 | 0.745-0.855 |
| C20:4 n-6 | 0.693 | 0.625-0.761 |

Table S2

The ROC curve analysis of fatty acids for episodic migraineurs and chronic migraineurs.

| **ROC curve analysis** | **AUC** | **95% CI** |
| --- | --- | --- |
| Logistic regression model | 0.769 | 0.670-0.867 |
| C16:0 | 0.725 | 0.621-0.828 |
| C20:4 n-6 | 0.706 | 0.611-0.801 |

Table S3

Multiple linear regression analysis of fatty acids with HIT-6 and MIDAS.

| **Scales** | **Fatty acids** | **B** | **SE** | **Beta** | **t** | ***P-*value** | **95%CI** | **R^2^** |
| --- | --- | --- | --- | --- | --- | --- | --- | --- |
| HIT-6 | C16:0 | 0.164 | 0.030 | 0.437 | 5.518 | <0.001 | 0.105-0.223 | 0.304 |
|  | C20:4 n-6 | 1.235 | 0.343 | 0.289 | 3.595 | <0.001 | 0.554-1.915 |  |
| MIDAS | C16:0 | 9.361 | 1.290 | 0.481 | 7.258 | <0.001 | 6.805-11.916 | 0.529 |
|  | C20:4 n-6 | 0.768 | 0.111 | 0.449 | 6.886 | <0.001 | 0.547-0.989 |  |

Β: the non-standardized coefficient, Beta: the standardized coefficient, R²: the determination coefficient, *p*<0.05 indicated statistically significant difference.
